# Prospects for surviving climate change in Antarctic aquatic species

**DOI:** 10.1186/1742-9994-2-9

**Published:** 2005-06-06

**Authors:** Lloyd S Peck

**Affiliations:** 1 Natural Environment Research Council, British Antarctic Survey, High Cross, Madingley Road, Cambridge CB3 0ET, UK

## Abstract

Maritime Antarctic freshwater habitats are amongst the fastest changing environments on Earth. Temperatures have risen around 1°C and ice cover has dramatically decreased in 15 years. Few animal species inhabit these sites, but the fairy shrimp *Branchinecta gaini *typifies those that do. This species survives up to 25°C daily temperature fluctuations in summer and passes winter as eggs at temperatures down to -25°C. Its annual temperature envelope is, therefore around 50°C. This is typical of Antarctic terrestrial species, which exhibit great physiological flexibility in coping with temperature fluctuations. The rapidly changing conditions in the Maritime Antarctic are enhancing fitness in these species by increasing the time available for feeding, growth and reproduction, as well as increasing productivity in lakes. The future problem these animals face is via displacement by alien species from lower latitudes. Such invasions are now well documented from sub-Antarctic sites.

In contrast the marine Antarctic environment has very stable temperatures. However, seasonality is intense with very short summers and long winter periods of low to no algal productivity. Marine animals grow slowly, have long generation times, low metabolic rates and low levels of activity. They also die at temperatures between +5°C and +10°C. Failure of oxygen supply mechanisms and loss of aerobic scope defines upper temperature limits. As temperature rises, their ability to perform work declines rapidly before lethal limits are reached, such that 50% of populations of clams and limpets cannot perform essential activities at 2–3°C, and all scallops are incapable of swimming at 2°C. Currently there is little evidence of temperature change in Antarctic marine sites. Models predict average global sea temperatures will rise by around 2°C by 2100. Such a rise would take many Antarctic marine animals beyond their survival limits. Animals have 3 mechanisms for coping with change: they can 1) use physiological flexibility, 2) evolve new adaptations, 3) migrate to better sites. Antarctic marine species have poor physiological scopes, long generation times and live on a continent whose coastline covers fewer degrees of latitude than all others. On all 3 counts Antarctic marine species have poorer prospects than most large faunal groups elsewhere.

## Background

A current major focus for science is the prospect of global climate change. It is now internationally accepted that we are in a period of global change, that for some measures such as atmospheric carbon dioxide content, is faster than at any time in recent geological history [1,2]. Much effort has been put into producing models of predicted change [1,3,4], and identifying the evidence for change. These models and their outputs have had profound effects on the science conducted and the focus of research in both physical and biological disciplines.

Environmental change poses a range of problems for species that vary between site and species. Abilities to cope with change differ from individual to species levels. Research into abilities to cope has focussed primarily on temperature change, but other environmental factors including humidity, precipitation and windflow patterns on land and ocean currents and local salinity in the sea are also likely to be affected [1,2]. We are clearly still in the early stages of understanding how biotas around the world will or can respond to change, but such evaluations are essential for the optimum management or mitigation of deleterious effects, both for mankind and the environment.

In this review I evaluate the major characteristics of Antarctic species and contrast the threats and abilities to cope with change for aquatic organisms from terrestrial and marine environments. Emphasis is placed on current environmental variation, physiological flexibility (where flexibility refers to the ability to cope either within immediate tolerance limits or to acclimate over a period of weeks or months), and future threats. If environmental conditions deteriorate organisms can respond by: (1) coping within their physiological tolerance range, or within their ability to acclimate; (2) evolving new characters to allow survival; and (3) migrating to sites where conditions have remained or become favourable [5,6]. The review ends with a general overview of the possible responses that organisms can employ to survive change, and how Antarctic species compare with communities elsewhere in these characteristics.

### Antarctic Freshwater Environments

**Figure 1:**
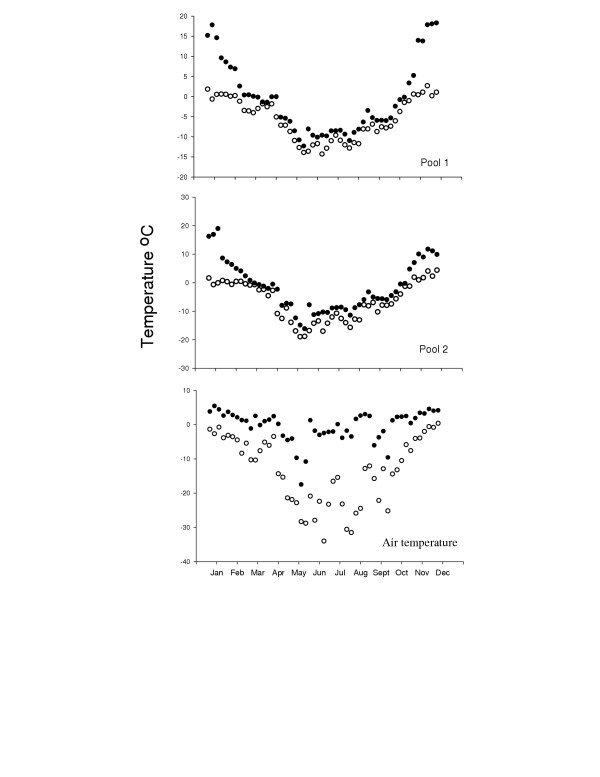
Water and adjacent air temperature from Jan to Dec 2002 inclusive for two pools containing dense populations of the fairy shrimp *Branchinecta gaini *on Anchorage Island, Ryder Bay, Adelaide Island (67°34'07"S, 68°07'30"W). Temperatures were logged at 5 min intervals, and are shown as weekly maxima (●) and minima (○). Time points indicated are mid-month.

More than 99.5% of continental Antarctica is covered by ice [7] that is, in places, over 4 km thick, and open areas are restricted to the continental margin and mountaintops. Even though there is significant melting of ice in some areas in summer, very few sites have habitable aquatic sites. However, in the maritime Antarctic, along the Antarctic peninsula and offshore islands, significant pools and lakes exist that are inhabited by microbial, algal and invertebrate communities, some of which can be substantial. These pools and lakes are usually seasonal, being frozen in winter, although some of the larger water masses only freeze at the surface. The extent of the period of open water, and temperatures that any of them experience in summer are a function of many factors including air temperature, altitude and aspect. Pools that sustain significant populations of fairy shrimps, *Branchinecta gaini*, occur near Rothera station on the Antarctic peninsula (Fig. 1). In these pools summer temperatures are positive, but fluctuate between 0°C and 20°C, whereas in winter temperatures fall to around -20°C, but are more stable. The fairy shrimps living there thus experience an annual temperature range of 40°C, or more, and in summer can experience daily temperature fluctuations of up to 20°C. Similar, or more extreme temperature regimes are common in maritime Antarctic terrestrial systems [8].

Ecological conditions in maritime Antarctic (which includes coastal regions of the Antarctic peninsula and several offshore islands [9]) freshwater environments vary markedly over short distances, with nutrients such as orthophosphate and ammonia, as well as water alkalinity varying by more than an order of magnitude between adjacent lakes [10]. Algal communities also vary markedly between localities, with Chlorophyll a concentrations varying by factors of 5 or more between lakes on Signy Island [10]. These marked variations between pools and lakes over short distances are the product of a range of factors including: aspect effects on insolation; small variations in temperature; hydrology and erosion in the catchment area; and in-lake biological activity and biogeochemical cycling.

Thus over the majority of the Antarctic continent there are very few sites where aquatic systems are available for colonisation. However, at the continental margins and in the maritime Antarctic significant numbers of pools and lakes exist where biological communities are established, and these environments are generally both cold and frozen in winter and highly variable in ecological characters.

### Physiological capacities of Antarctic Freshwater species

Species inhabiting pools and lakes in Antarctica exhibit great physiological flexibility in coping with variations in environmental temperature. *B. gaini *populations can survive temperatures up to 10°C for 1 week with no mortality, and temperatures up to 25°C for 48 h with only 50% mortality [11]. Oxygen consumption rates also rise in an expected fashion with temperature, doubling for every 10°C rise in temperature in this species, with rates at 20°C four times higher than those at 1°C [11]. This indicates a metabolic scope for *B. gaini *of at least × 4 (where a metabolic, or physiological scope refers to the maximum ability to raise a function over the minimum required for long-term survival). Ventilation rate on the other hand only increased by a factor of 2.2 between 1°C and 20°C. This mismatch between capacity to raise ventilation rate and oxygen consumption suggests that, although oxygen delivery does not appear limiting between 1°C and 20°C, the upper lethal limit may be dictated by an inability to raise oxygen supply mechanisms at a sufficient rate, as seen in marine ectotherms [12].

Summer temperature variations are survived as adults. Adults cannot, however, survive freezing, and winter periods are passed as eggs, or cysts, which remain unfrozen to temperatures around -25°C. Antarctic terrestrial species, predominantly Collembola and Acari survive even wider temperature fluctuations as adults, using antifreezes, cryoprotectants and supercooling to maintain viability at temperatures down to -30°C, or below [13]. These groups often endure annual temperature ranges in excess of 50°C [13]. Terrestrial invertebrates in Antarctica appear to have very wide physiological flexibility, and show cold adapted metabolic rates that are faster than metabolic rates of similar species from lower latitudes at any given temperature [14]. Growth rates are also reported to show some temperature compensation, and the optimum temperatures for feeding and growth are low [15].

Terrestrial and freshwater species, therefore, exhibit large physiological flexibility, giving them the ability to function normally at temperatures between 0°C and 15–20°C. They also possess adaptations allowing short-term survival in the range -25°C to +25°C for freshwater species and -30°C to +30°C for some terrestrial Acari and Collembola.

### Antarctic marine environments

**Figure 2:**
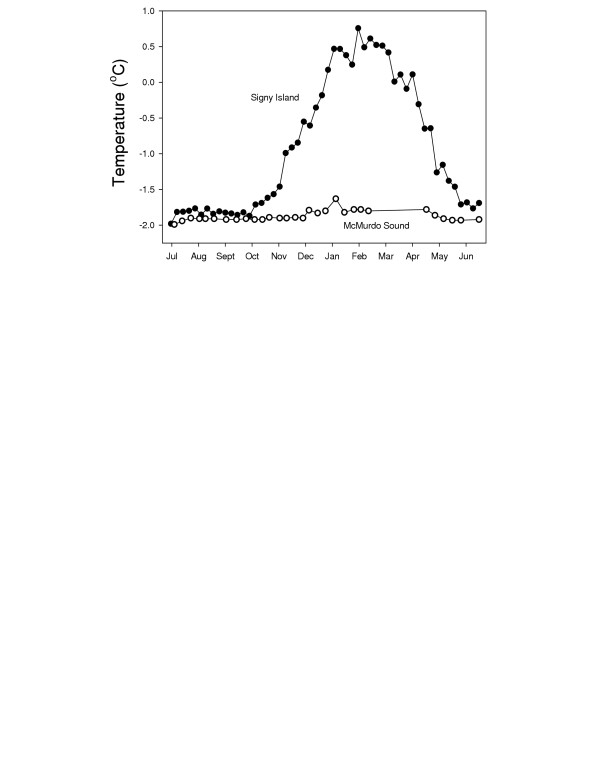
Annual cycle of seawater temperature for two sites in Antarctica. (●) 10 m depth, Orwell Bight, Signy Island (10 year mean values); (○) McMurdo Sound. Figure updated from [16].

The Southern Ocean is characterised by low but highly stable temperatures. At the most variable sites, such as Signy Island in the maritime Antarctic, temperatures usually range between -1.8°C in winter and around +1.0°C in summer (Fig 2). At the highest Antarctic sites, typified by McMurdo Sound, temperatures range between-1.7°C and -2.0°C yearly [16], although recent data suggest temperatures may be slightly more variable than this at McMurdo, with temperatures approaching -0.5°C in some years [17]. Thus the total annual fluctuation in sea temperature rarely exceeds 3°C in the region of the Antarctic Peninsula and Scotia Sea and is half this or less at high Antarctic (continental coast), making this one of the most thermally stable environments on earth. Temperatures have been low and stable around Antarctica for a long evolutionary period, at least 10 million years.

Other characters of the marine environment vary markedly with season around Antarctica. Annually light varies between no direct sunlight and overall radiative loss from the surface to 24 h direct sunlight and incident radiation levels at or above tropical values over a 24 period in summer. Between 10 and 15 million km^2 ^of sea-ice forms in winter and melts in summer. These factors result in intense seasonality of phytoplankton productivity, especially in nearshore waters. Blooms around Signy Island, South Orkney Islands (60°43'S, 45°36'W) and Rothera Point, Adelaide Island (67°36'S, 68°12'W) are both restricted to approximately 2–3 month periods and at peak reach chlorophyll standing stock levels in excess of 25 mg Chl a m^-3 ^[18,19], values that would be high for any site on earth.

In the open ocean productivity is often associated with the edge of the sea-ice, and a significant portion of overall oceanic productivity occurs in these areas [20]. Micro-algal productivity associated with sea-ice can also be extensive [21], as can blooms of benthic communities on the seabed [22]. When taken together these 3 areas of productivity can be a more significant source of resource supply to secondary consumers than open ocean blooms in the Southern Ocean.

### Physiological capacities of Antarctic marine species

**Figure 3:**
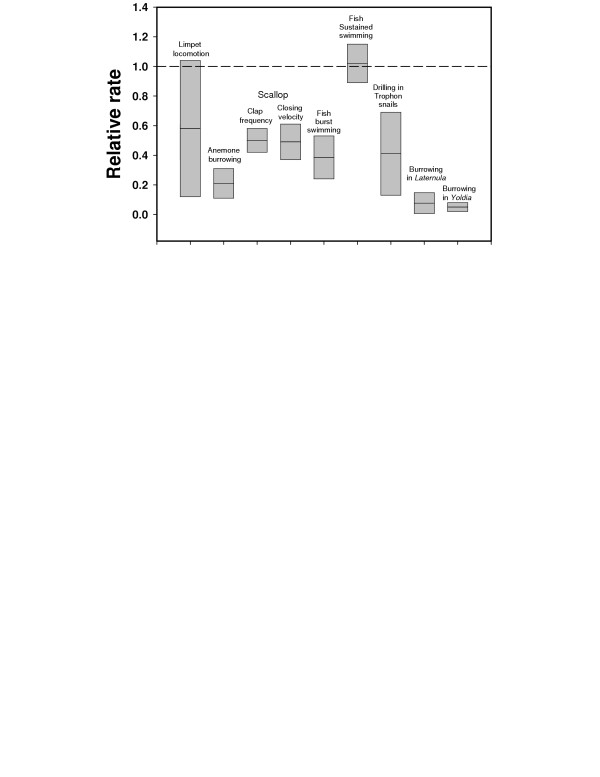
Rates of accomplishing various activities for a range of Antarctic marine invertebrates compared with related or ecologically similar temperate species. The hatched line indicates representative rates for temperate species and is set at a value of 1. Boxes show the range of values, with the midline indicating the mean. Figure reproduced from [24].

**Figure 4:**
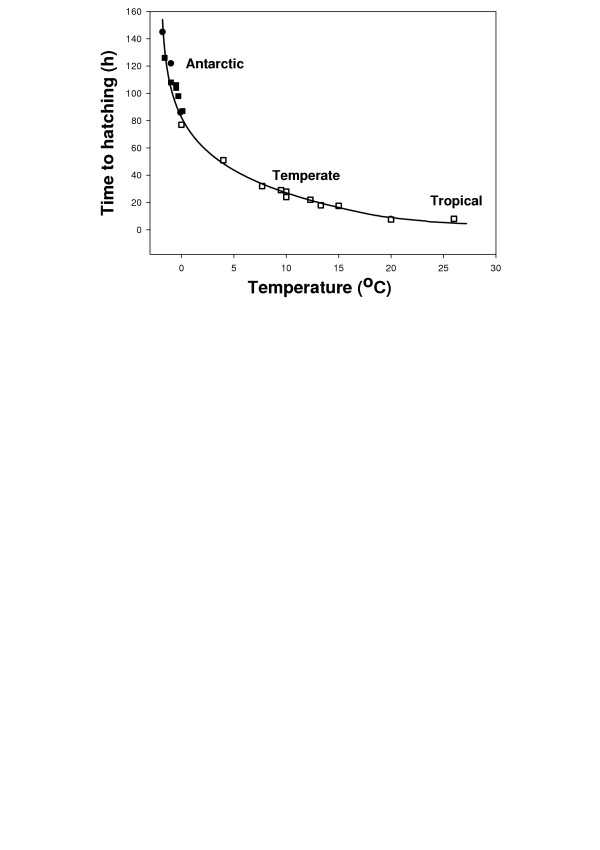
Time to hatching for echinoderm embryos from Antarctic, temperate and tropical sites. □ = temperate and tropical echinoderms [25]; ● = Antarctic species [25]; ■ = Antarctic species [31]. Figure modified from [25, 31].

Antarctic marine ectotherms are characterised by low rates of growth, development, metabolism and activity with little or no evidence of temperature compensation [23,24] (Fig. 3). In general growth rates are 2–5 times slower in Antarctic species compared to similar temperate organisms, and larval development is 5–10 times slower, with echinoderm embryos taking up to 150 h from fertilisation to hatching in Antarctica [23,25] (Fig. 4). The metabolic rate of adult animals is lowered by a factor of 2 to 3 times for every 10°C drop in temperature compared with lower latitude species [26,27], and metabolic rates of larvae may be even more affected by temperature than adult animals, being around 10 times lower in larvae of Antarctic gastropod molluscs than temperate species [28].

As well as exhibiting low rates for most life history characters, Antarctic marine species are also highly stenothermal, dying in experiments when temperatures are raised to between +5°C and +10°C [26]. There is now strong evidence that these low upper temperature limits are set by a limitation to aerobic scope, or poor capacity to raise metabolic rates to perform work [12,29,30]. In the bivalve mollusc *Laternula elliptica *oxygen consumption and heartbeat rate rise with temperature between 0°C and 9°C, where both collapse and animals begin to die. Both oxygenated and deoxygenated blood is obtained from samples at 0°C, 3°C and 6°C, but no oxygenated blood is present at 9°C, and death ensues from a deficiency in oxygen supply to the tissues [32]. At around 5°C anaerobic end products of metabolism such as succinate and acetate begin to accumulate, indicating a long-term physiological limit, termed the critical limit [29].

That Antarctic marine species have poor capacities to raise metabolism for work has been shown for several species. The brachiopod *Liothyrella uva *can only elevate metabolism by × 1.7 over resting rates at 0°C when temperature is raised and the limpet *Nacella concinna, *by a factor of 2.6 [33]. In bivalves the value for *Limopsis marionensis *× 1.8 [34], and for *Laternula elliptica *is × 2.7 [32]. These values compare with the factor of over 4 that the Antarctic freshwater fairy *s*hrimp *B. gaini* raises its rate of oxygen consumption with increased temperature. One difference here is that in experiments on the marine species temperatures were only raised to between +4°C and +10°C, where animals died, compared to the 25°C for *B. gaini *.

**Figure 5:**
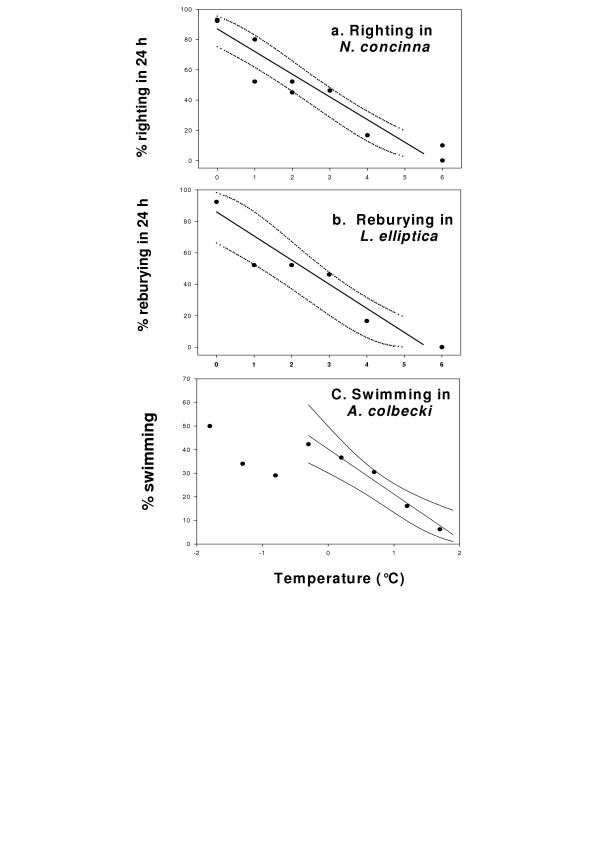
The effect of temperature on activity in Antarctic marine species. **a**. The proportion of animals in a population of the limpet *Nacella concinna *capable of righting when turned over. **b**. The proportion of the infaunal bivale *Laternula elliptica *capable of reburying when removed from sediment. **c**. The proportion of scallops, *Adamussium colbecki *capable of swimming. A regression was fitted to data for temperatures above 0.3°C, where a clear relationship exists. Below this value no temperature restriction is apparent. In all 3 graphs regressions were fitted following arcsin transforms of the square root of % values. Dotted lines indicate 95% confidence intervals. Figure modified from [35].

Limited aerobic scopes have further consequences for Antarctic marine ectotherms. Their ability to perform activity or work is severely temperature restricted. Thus, 50% of individuals of populations of burrowing bivalves, *Laternula elliptica *lose the ability to bury when temperatures are raised to 2.5°C, there is complete loss of burrowing ability in all animals at 5°C, and large reproductive individuals lose the ability to bury first [35] (Fig. 5). Populations of the limpet *Nacella concinna *similarly lose the ability to right themselves when turned over, with 50% loss of ability at temperatures between 2°C and 2.5°C. The scallop *Adamussium colbecki *is even more severely constrained, with no animals capable of swimming when temperatures are raised to 2°C [35]. Essential biological functions are, therefore rapidly lost with rising temperature in Antarctic marine species. Such limited capacity will clearly have marked effects on abilities to obtain and process food, and to reproduce, and hence animal fitness. This factor may be more important ecologically in terms of survival of populations and species than the physiological limits described earlier, although the physiological and ecological restrictions caused by extreme temperature sensitivity will be intimately linked. Current maritime Antarctic summer sea temperatures are usually around 1°C. Reproductive sized *L. elliptica *and *N. concinna *lose the ability to perform significant activities at, or below 2.5°C, only 1.5°C above current summer maxima, when animals need to exploit the short phytoplankton bloom. Scallops are even more tightly constrained, with all individuals losing the ability to perform important biological activities at temperatures less than 1°C above current summer maxima. These data are all for bivalve molluscs. However, the effect is likely to be widespread and suggests that a 1°C rise in summer sea temperatures around Antarctica could have severe ecological consequences for populations and communities of marine ectotherms.

### Predicted environmental change and potential consequences

#### Freshwater habitats

The Antarctic peninsula is currently one of the fastest changing environments on earth, with air temperatures having risen by over 2°C in the last 50 years at some sites [4]. Aquatic environments may be changing even faster than this, with temperatures in lakes on Signy Island having risen by nearly 1°C through the 1980's and 1990's, at a site where air temperatures rose by around 1°C in 40 years [10]. Lakes and pool temperatures are changing faster than local air temperatures because of an amplification effect of reduced ice cover throughout the year. As ice cover decreases a longer period of the year is available for sunlight to penetrate to sediments, which are then heated and higher temperatures are maintained through the winter [10]. Current models predict that subantarctic and maritime Antarctic terrestrial sites are likely to warm significantly over the next 100 years, by possibly as much as 5°C [36]. The result of this will be to make sites where freshwater invertebrates currently exist better habitats for colonisation, and populations of species like the fairy shrimp *B. gaini *are likely to expand. New sites further south are also likely to become available for colonisation, as pools further south become unfrozen and algal mats build up. The overall consequence, therefore for freshwater species will be an extension of range further south and an improvement of habitat increasing overall population sizes.

As seen earlier species inhabiting terrestrial and freshwater sites have wide physiological flexibilities to cope with environmental changes of the type predicted. The major problem facing the limited number of species currently inhabiting Antarctic freshwater environments is displacement by alien species that are not capable of colonising current habitats. Alien invasions of subantarctic island sites are well documented [37,38], and many of the invasions have used humans as vectors. This is clearly the case for both rats and cats, as well as grasses and insects on a range of islands [37]. However, invasions using natural vectors have occurred, and under predicted future climate change, at some stage conditions will have reached levels where alien species can invade, and current endemics will be displaced. The likely outcome is that current endemic species will move south to areas where alien species are not capable of establishing populations, and lower latitude maritime Antarctic sites will be colonised by alien species, or mixed communities of current endemics and alien invaders.

#### Marine environments

There is little evidence that sea temperatures are changing around Antarctica, although one report has indicated Antarctic mid water increased in temperature by 0.17°C between the 1950's and the 1980's [39]. Current models predict global sea temperature will rise on average by 2°C in the next 100 years [1,36]. However, there will be marked regional variation, with some areas warming more than 2°C, and others not changing, or possibly cooling following the movement of large-scale ocean currents. Temperature changes in polar oceans are particularly difficult to predict, because of a lack of knowledge concerning how sea-ice is likely to change, and the effects this will have on the underlying water temperatures.

Other factors that are likely to change with rising temperatures are sea-ice and ice shelf cover and productivity. Satellite data indicate sea-ice extent has not changed markedly over the last 25 years [40], and models vary in their predictions of sea-ice cover over the next 100 years. The IPCC panel prediction was that there is likely to be a decrease of around 25% in sea-ice extent over the next 100 years [41]. As significant productivity is associated with the ice edge, a reduction in ice cover will reduce the extent of these blooms, reducing overall productivity.

Responses that enhance survival when the environment changes fall into three main categories, (1) using physiological capacities or scopes, (2) adapting to new conditions, or (3) migrating to areas that provide a better environment. From the earlier analysis, the Antarctic marine fauna is characterised by limited physiological capacities, and has a poor ability to cope with change using physiological capacities, possibly the poorest of any fauna so far described. The Antarctic marine fauna is characterised by slow growth, increased longevity and deferred maturity [23]. Of the species studied, several live in excess of 40–50 years [23,35,42]. Generation times are often in the range 10–20 years. Abilities to produce new adaptations to changing conditions are, therefore, poor. In excess of these considerations most continents have long coastlines covering many degrees of latitude and this, in many cases, allows for migration to more hospitable conditions when environments deteriorate. The coastline of Antarctica covers relatively few degrees of latitude, and less than any other continent. Species inhabiting the Southern Ocean seabed, therefore, have less scope to migrate away from poor conditions than faunas elsewhere. The combination of very poor physiological capacities, with extended life histories producing slow rates of adaptation and restricted available dispersal ranges make Antarctic marine species amongst the least capable of responding to environmental change on earth.

## Conclusion

Terrestrial Antarctic species are adapted to very variable conditions and have good prospects for surviving in changing environments. It is likely that species diversity, community biomass and complexity will increase in coming years. There is a strong likelihood that new species will eventually appear on the Antarctic continent as warming continues, and these new species could displace the current endemic groups.

Marine species face a different problem. They have evolved in an environment with possibly the lowest temperature variation on Earth, and have lost the ability to cope with temperature change. A rise in summer sea temperatures of only 2°C could potentially compromise survival for many Antarctic benthic species. They may be one of the most vulnerable faunal groups to environmental change on Earth.
